# Prescribing patterns in patients with chronic obstructive pulmonary disease and atrial fibrillation

**DOI:** 10.1515/med-2023-0864

**Published:** 2023-11-30

**Authors:** Kuang-Ming Liao, Pei-Jun Chen, Chung-Yu Chen

**Affiliations:** Department of Internal Medicine, Chi Mei Medical Center, Chiali, Taiwan; Department of Nursing, Chi Mei Medical Center, Chiali, Taiwan; Master Program in Clinical Pharmacy, School of Pharmacy, Kaohsiung Medical University, 100 Shih-Chuan 1st Road, Sanmin District, Kaohsiung 80708, Taiwan; Department of Pharmacy, Kaohsiung Medical University Hospital, Kaohsiung, Taiwan; Department of Medical Research, Kaohsiung Medical University Hospital, Kaohsiung, Taiwan

**Keywords:** atrial fibrillation, chronic obstructive pulmonary disease, non- vitamin K antagonist oral anticoagulants, prescribing pattern, proportion of days covered

## Abstract

Patients with chronic obstructive pulmonary disease (COPD) had higher risk of atrial fibrillation (AF). The treatment of AF includes medicines to control heart rate and reduce the risk of stroke, and procedures such as cardioversion to restore normal heart rhythm. To reduce the stroke, patients with AF may prescribe some type of antithrombotic medication (such as warfarin, one of the new non-vitamin K antagonist oral anticoagulants [NOACs] – dabigitran, apixaban, rivoraxaban, or edoxaban) or maybe aspirin. The aim of our study was to exam the prescription pattern in patients with COPD and AF. We selected COPD population in Taiwan older than 40 years and less than 90 years old with an COPD diagnosis at least two outpatient claims or at least one inpatient claim coded and also need at least one prescription of bronchodilators. We followed this COPD cohort until they have AF and their prescription pattern. We included 267,740 patients with COPD who meet the inclusion and exclusion criteria and 6,582 patients concomitant with COPD and AF. The mean age was 75 years, and about 77% of the patients were older than 70 years. Three-fourths of patients with COPD were male. The common comorbidities were hypertension (17.58%), diabetes (7.47%), ischemic heart disease (4.66%), and dyslipidemia (3.68%). we found that most patients received aspirin which accounting for 31%, followed by coumadin (8.22%) and clopidogrel. Prescribing NOAC within 30 days after AF diagnosis was low in patients with COPD and the percentage of NOAC usage was also lower than warfarin.

## 1 Introduction

Atrial fibrillation (AF) is a commonly occurring irregular and often rapid heart rhythm disturbance. The prevalence of AF is as high as 2% in adults and increases with age. AF is not only associated with an increased risk of mortality but also responsible for 20–30% of all strokes [1]. There are some potential mechanisms between AF and chronic obstructive pulmonary disease (COPD). Many factors, including deoxygenation, hypercapnia, systemic inflammation, production of reactive oxygen species, and gas trapping and hyperinflation, result in pulmonary hypertension and increased pulmonary vascular resistance, diastolic dysfunction, and increased arrhythmogenicity due to respiratory drugs contribute to AF in COPD patients [2]. The adjusted hazard ratio (HR) of AF in patients with COPD was 2.23 (95% confidence interval [CI] 1.98–2.51) compared to patients without COPD [3]. COPD is also an independent risk factor for AF progression and recurrence [4]. AF has been shown to have a negative impact on COPD patients during hospitalization. COPD patients with AF had a higher risk of hepatic dysfunction and respiratory failure than those without AF [5].

The risk of stroke is high in patients with AF and COPD, but patients with COPD generally have many comorbidities, and the bleeding risk is higher in Asian populations than in Western countries. Physicians’ decisions to follow prescription guidelines seem to be influenced by many factors; however, real-world prescribing patterns in COPD patients with AF are unknown.

To our knowledge, no study has investigated the prescribing patterns in COPD patients after diagnosis with AF. The aim of this study was to use a claims database in Taiwan to elucidate the prescribing patterns in patients with COPD after AF diagnosis.

## 2 Materials and methods

### 2.1 Data source

This was a retrospective cohort study. We used data from the National Health Insurance Research Database (NHIRD) collected from 2002 to 2015. Taiwan launched a single-payer National Health Insurance program on March 1, 1995, and more than 99.9% of Taiwan’s population was enrolled.

This database contains a continuous record of hospitalizations, outpatient care, prescribed medicines, outcomes, and expenditures, as well as the details of the orders. All prescription medicines, including drug type, dosage, and dispensing date, are also recorded in the NHIRD. This study utilized health records that were deidentified so that the researchers were unable to view personal content, and the study was approved by the Institutional Review Board (IRB) of Kaohsiung Medical University Hospital (KMUH-IRB-EXEMPT (II)-20170003). Informed consent for this retrospective cohort study was waived due to the encryption of the claims data. This study was approved by the research ethics committee and performed in accordance with the ethical standards of the Declaration of Helsinki.

### 2.2 Study population

The study population in Taiwan consisted of patients between 40 and 90 years old with a COPD diagnosis (International Classification of Diseases-9-Clinical Modification [ICD-9-CM]: 490–492, 496) who had at least two outpatient claims or at least one inpatient claim in the database. The patients also had at least one prescription for a bronchodilator, including a long-acting beta-agonist (LABA), long-acting muscarinic antagonist (LAMA), LABA/LAMA, or fixed-dose LABA and an inhaled corticosteroid.

We excluded patients with a diagnosis of asthma, lung cancer, lung transplant, or who died 30 days after their COPD diagnosis. We followed this COPD cohort until they developed AF (ICD-9-CM code: 427.3). The date of the index AF diagnosis was defined as the index date in the study population. We excluded patients with a history of AF, patients who used anticoagulants before AF, or patients who died on or before the index date. COPD moderate exacerbations were defined as treatment using systemic steroid and/or antibiotics at exacerbation. COPD severe exacerbations require emergency room visits or hospitalization.

### 2.3 Outcomes and baseline characteristics

We followed all patient prescriptions for 30 days after the AF diagnosis. We also followed the COPD and AF patients from the index date to the day of death, the date of December 31, 2016, or until withdrawal from the NHIRD. Baseline comorbidities were identified from 2 outpatient diagnoses or 1 inpatient diagnosis 1 year before the index date. Comorbidities included diabetes mellitus (ICD-9-CM: 250), hypertension (ICD-9-CM: 401–405), dyslipidemia (ICD-9-CM 272.0–272.4), ischemic heart disease (ICD-9-CM: 410–414), cerebrovascular disease (ICD-9-CM: 430–434), end-stage renal disease (ICD-9-CM: 585), and liver disease (ICD9-CM: 570, 571, 572, 573). Demographic characteristics were evaluated in the COPD patients with AF and included sex, age group, and urbanization (level 1 [highest] to 4 [lowest]). We surveyed the anticoagulant and platelet inhibitors, which included aspirin, clopidogrel, coumadin, dipyridamole, ticlopidine, cilostazol, eptifibatide, tirofiban, abciximab, ticagrelor, dabigatran, rivaroxaban, apixaban, and edoxaban.

### 2.4 Statistical analysis

Continuous variables were compared between the case and control groups with Student’s *t*-test. Categorical variables are presented as numbers and percentages, and the differences between the cases and controls were compared with the chi-square test. We used the proportion of days covered (PDC) to evaluate medication adherence in the patients with COPD and AF; PDC was defined as the proportion of days in the observed period “covered” by an anticoagulant and platelet inhibitor in its therapeutic category. The measurement period for adherence was 1 year after the index date or the date an outcome occurred, the date of death, or December 31, 2016. Patients were divided into high PDC (≥80%) and low PDC (<80%) groups according to adherence. COPD exacerbations were divided into moderate or severe exacerbations. Moderate exacerbations were defined as requiring treatment with oral corticosteroids or antibiotics or both, and those additionally requiring hospitalization or an emergency room visit were considered severe. COPD exacerbation information was collected during 1 year before the index date for all patients.

A *P*-value of <0.05 was considered to be statistically significant in all statistical analyses. All data processing and statistical analyses were performed using SAS^®^ software version 9.4 (SAS Institute Inc., Cary, NC, USA).

## 3 Results

**Figure 1:**
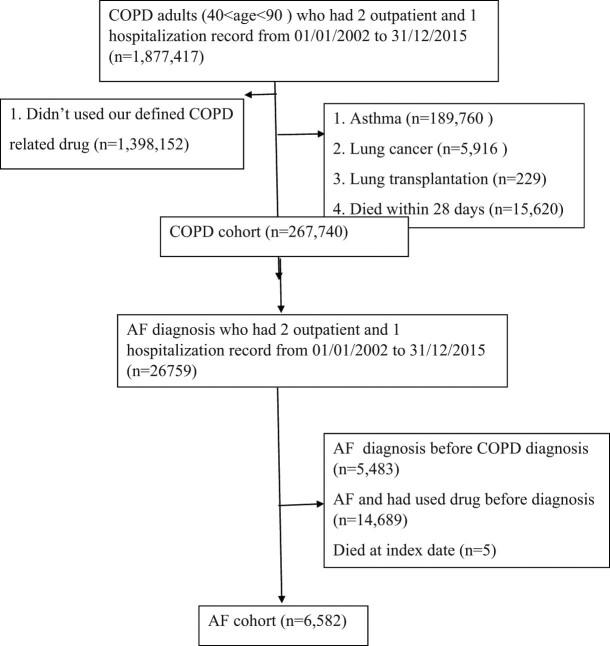
Flowchart showing the patient selection process for analysis.

The baseline characteristics of the COPD patients with AF in our study are shown in Table 1. We included 267,740 patients with COPD who met the inclusion and exclusion criteria and 6,582 patients with concomitant COPD and AF. Patient selection is illustrated in a flowchart (Figure 1). The mean age was 75 years and approximately 77% of the patients were older than 70 years. Three-fourths of the patients were male. The most common comorbidities were hypertension (17.58%), diabetes (7.47%), ischemic heart disease (4.66%), and dyslipidemia (3.68%).

**Table 1:** Demographic characteristics of and comorbidities in COPD patients with AF

	COPD and AF (*N* = 6,582)	(%)
**Age stratification (%)**		
40–49	51	(0.77)
50–59	359	(5.45)
60–69	1,096	(16.65)
≥70	5,076	(77.12)
**Sex (%)**		
Male	5,070	(77.03)
Female	1,512	(22.97)
**Comorbidities**		
Diabetes	491	(7.46)
Hypertension	1,157	(17.58)
Dyslipidemia	242	(3.68)
Ischemic heart disease	307	(4.66)
Cerebrovascular disease	79	(1.20)
Liver disease	139	(2.11)
End-stage renal disease	85	(1.29)

Table 2 shows the prescribing patterns in COPD patients after the diagnosis of AF. In our claims database, we found that most patients received aspirin, accounting for 31%, followed by coumadin (8.22%) and clopidogrel.

**Table 2:** Prescribing patterns within 30 days after AF diagnosis

	COPD and AF (*N* = 6,582)	%
	*n*	
Bokey	2,048	31.12
Plavix	329	4.99
Warfarin	541	8.22
Dipyridamole	264	4.01
Ticlopidine	110	1.67
Cilostazol	11	0.17
Tirofiban	2	0.03
Ticagrelor	5	0.08
Dabigatran	110	1.67
Rivaroxaban	103	1.56
Apixaban	8	0.12
Edoxaban	2	0.03

Table 3 shows that the adherence rates after COPD patients were diagnosed with AF for the first year. We divided patients into high PDC (≥80%) and low PDC groups (<80%). We also classified COPD exacerbation within the first year according to our definition. More than 63% of patients had moderate exacerbation more than twice. A total of 64% of patients did not present with severe exacerbation.

**Table 3:** Adherence in patients with COPD and AF

Variable	Low PDC (<80%)		High PDC (≥80%)		*P*-value
	*N* = 6,004		*N* = 578		
	*n*	(%)	*n*	(%)	
**Moderate COPD exacerbation**					
0	1,487	(24.77)	135	(23.36)	0.4523
1	723	(12.04)	68	(11.76)	0.8448
≧2	3,794	(63.19)	375	(64.88)	0.4213
**Severe COPD exacerbation**					
0	3,823	(63.67)	391	(67.65)	0.0573
1	1,067	(17.77)	114	(19.72)	0.2428
≧2	1,114	(18.55)	73	(12.63)	0.0004
**Sex**					
Female	1,408	(23.45)	104	(17.99)	0.0029
Male	4,596	(76.55)	474	(82.01)	
**Age stratification**					
40–49	44	(0.73)	7	(1.21)	0.2104
50–59	340	(5.66)	19	(3.29)	0.0163
60–69	992	(16.52)	104	(17.99)	0.4304
≥70	4,628	(77.08)	448	(77.51)	0.8156
**Comorbidities**					
Diabetes	455	(7.58)	36	(6.23)	0.2381
Hypertension	1,053	(17.54)	104	(17.99)	0.7838
Dyslipidemia	222	(3.70)	20	(3.46)	0.7721
Ischemic heart disease	277	(4.61)	30	(5.19)	0.5300
Cerebrovascular disease	74	(1.23)	5	(0.87)	0.4384
Liver disease	133	(2.22)	6	(1.04)	0.0601
End-stage renal failure	80	(1.33)	5	(0.87)	0.3418

PDC: proportion of days covered.

## 4 Discussion

To our knowledge, this is the first study to investigate prescribing patterns in COPD patients with AF. In our study, we found that many COPD patients with AF were not prescribed a nonvitamin K‐dependent oral anticoagulant (NOAC) or warfarin after diagnosis. The percentage of patients who received an NOAC or warfarin within 30 days after the diagnosis of AF was low.

A previous observational study was performed in a single hospital to determine which factors contributed to poor medication adherence in AF patients treated with oral anticoagulants in a real-world clinical setting. They found that poor medication adherence was related to emotional response, younger age, no history of warfarin treatment, and long anticoagulant exposure [6].

Dabigatran was the most commonly prescribed NOAC among Taiwanese patients with COPD and AF during our study period, followed by rivaroxaban. This trend was also observed by Olesen et al. in the Danish nationwide administrative registry study, as dabigatran was the first NOAC to enter the market in Denmark [7]. In Taiwan, rivaroxaban entered the market in July 2009, followed by dabigatran in June 2011, apixaban in April 2013, and edoxaban in February 2016. The market share of NOAC might be due to the market-release time, prescription preference, or characteristics of treatments and included patients. Our study included only COPD patients with AF, and this inclusion criterion might have affected the prescribing trend. Further investigations with longer follow-up periods are needed to understand the prescribing trend and impact of the market entry of several kinds of NOACs.

The reimbursement criteria of Taiwan health insurance may influence prescribing patterns. NOACs were not covered for all AF patients in Taiwan. They were limited to only patients with nonvalvular AF who met one of the following conditions: (1) patients who had a stroke or systemic embolism; (2) patients for whom the left ventricular ejection fraction was less than 40%; (3) patients with symptomatic heart failure (prior to case closing, failure was classified as Grade 2 or higher according to the New York Heart Association); (4) patients older than 75 years (inclusive); and (5) patients aged 65–74 years with diabetes, hypertension, or coronary artery disease. Thus, caution is needed when interpreting our study results; in addition, it is not appropriate to extend the study findings to all NOAC users.

Nevertheless, understanding this special population, which includes patients with AF unsuitable for NOACs or warfarin, is still important. An easy bleeding tendency was observed in our previous study. A large proportion of patients with AF received Bokey or Plavix in the NOAC era in Taiwan. However, as time goes by, these Bokey or Plavix users could become unsuitable for these medications (e.g., increased risk of death, hospitalization, or stroke) [8,9,10,11]. Thus, understanding issues in this population is important, and this study provides insight into potentially inappropriate anticoagulation therapies in patients with COPD and AF.

A previous study showed that concomitant use of contraindicated drugs can affect the bleeding risk or treatment effectiveness by modifying the blood concentration of NOACs [12]. In our analysis, the concomitant use of enoxaparin and warfarin was relatively infrequent. Both drugs have the potential to increase bleeding risk. A previous study found that some patients prescribed dabigatran may concomitantly receive low-molecular weight heparin [13]. Thus, a medication monitoring system to detect such contraindicated combinations is warranted to reduce the risk of bleeding.

We did not find significant differences in the medication possession ratio among the three NOACs, and we supposed that compliance with the NOACs was high. Unlike warfarin, NOACs are associated with easy administration (oral formulations) and the absence of the need for careful monitoring in the hospital. Another study showed that adherent patients were older than nonadherent patients [14]. These results were consistent with our findings.

It is important for physicians to pay attention to first-episode AF, which is said to occur when arrhythmia is diagnosed for the first time, irrespective of the preceding or subsequent clinical course. In our study, patients with first-episode AF accounted for almost half of those admitted to the hospital. Just over 50% of these patients were discharged with AF without receiving anticoagulants. The duration between AF diagnosis and receiving a NOAC was more than 30 days.

However, therapeutic decisions cannot be delayed in patients who present with first-episode AF and who are discharged with sinus rhythm, a situation that occurred in 36 of the 200 (18%) patients admitted to the cardiology department. An approach to initiating such treatment in first-episode AF needs to be established. It cannot be argued that this patient population has a low thromboembolic risk profile, as this population had many risk factors. Recent results from our group suggest that insurance plans with good benefits (and generally low copayments) may be associated with increased odds of NOAC prescription [15]. Further characterization of these factors will help researchers better understand the drivers of AF treatment choice by healthcare providers.

Angel et al. [16] surveyed 4,427 patients admitted to their hospital with a history of AF between 2016 and 2018 and also found that 1,746 (39.4%) patients are lacking (no treatment despite indication) anticoagulant therapy among patients with AF. Up to 40% of patients with AF do not receive anticoagulant therapy. Our study evaluation of oral anticoagulant treatment reveals that the overall adherence rate was approximately 60% and is similar to that reported from a large American database by Marzec et al. [17] This is possibly the reason that antiplatelet agents were considered an alternative choice to oral anticoagulant agents, although this has long been proven to be ineffective to prevent ischemic stroke [18,19]. Unfortunately, this trend is still observed in a previous study, especially among older patients [20]. Our data further support the trend suggested in previous studies: physicians often choose to avoid or under-treat with oral anticoagulants [21,22,23,24], especially in the elderly and fragile population, potentially due to a perceived higher risk of bleeding than of ischemic stroke.

A prior study has highlighted age as an important factor impacting adherence to hypertension medication within the general population. Specifically, the study found that patient age was the sole factor associated with adherence, revealing that individuals under the age of 55 exhibited notably lower adherence compared to those aged 55–64 [25]. However, the available evidence on the role of age in predicting medication adherence presents a mixed picture. Existing literature consistently suggests that younger age tends to have a lower adherence [26]. On the other hand, when it comes to the elderly, a study proposed that older age is linked to better medication adherence [27]. Nevertheless, some conflicting findings have emerged, indicating that advancing age may negatively affect adherence among older individuals [28]. It is important to note that elderly patients may contend with additional challenges that impact their adherence, such as visual, auditory, and memory impairments. Moreover, cognitive decline or physical limitations, like difficulty swallowing tablets, handling small pills, distinguishing colors, or reading drug labels, can complicate adherence [29].

Furthermore, older adults may exhibit a heightened concern for their health compared to younger patients, potentially leading to involuntary non-adherence in many cases. Therefore, with adequate support from healthcare providers or family members, older individuals may be more likely to adhere to their medication regimen [26].

Additionally, four variables have been identified as factors that can categorize individuals as having either low or high medication adherence. These variables include health beliefs, disease duration, social support, and self-efficacy [30].

In our study, we found that there is statistically significant lower adherence in those aged 50–59 years and the adherence was increased with age and this may likely relate to awareness of the essential elements of the disease and raising awareness about the disease.

A systematic review and meta-analysis to evaluate the risk of stroke among COPD patients. There is a significantly increased stroke risk among COPD patients (HR, 1.30; 95% CI, 1.18–1.43). In subgroup analyses stratified by stroke subtype, study quality, and adjustment by socioeconomic status, the association between the risk of stroke and COPD was robust. [31]

COPD coexisting stroke will increase the burden on their caregivers, who are prone to adverse mental health and depression when looking after COPD or stroke patients [32,33]. Stroke may also result in lung function declined due to impaired cough and weakness of respiratory muscles and lead to high risk of pneumonia [34]. Accordingly, Patients had ischemic stroke and coexisting COPD significantly more often experienced cardiac and pulmonary complications, as well as delirium following stroke. The mortality was also higher in stroke patients with co-existing COPD after a long-term follow-up [35]. AF also increased the risk of stroke in patients with COPD and it is a great advantage of NOAC or warfarin treatment in patients with AF and COPD.

### 4.1 Limitations

This study has several limitations. First, the data used in our study did not include laboratory data (e.g., renal function) or body weight data, meaning that we could not evaluate whether the NOAC dose was appropriately reduced or not. Second, we could not determine whether the patients received all medications prescribed to them, which is one of the inherent limitations of studies using claims data.

Third, as is the case for all observational studies, potential unidentified confounders may exist. For example, our data source could not ascertain the AF subtype (e.g., permanent or paroxysmal) or symptom severity. We could not adjust for individual‐level socioeconomic status because the claims data source does not include details such as patient household income or education status. In our study, patients from 2002 and 2015 were included for observation, but NOAC did not enter the Taiwan market until 2009. The percentage of NOAC usage was underestimated while anti-platelet usage was overestimated and there is a bias between the percentage of NOAC and warfarin.

## 5 Conclusion

The prescription rate in patients with COPD and AF was low, and the percentage of NOAC usage was lower than that of warfarin usage. The reason for the low prescription rate needs further investigation.
